# Successful inguinal interstitial brachytherapy in metastatic cervical carcinoma: a case report

**DOI:** 10.3389/fonc.2023.1330681

**Published:** 2024-01-15

**Authors:** Yi Qin, Ping Guan, Dan Li, Huailin He, Wenfeng He, Longjing Tan, Xiangyu Deng, Bizhen Liao, Qinglian Wen, Zhenhua Zhang

**Affiliations:** ^1^ Department of Oncology, The Affiliated Hospital of Southwest Medical University, Southwest Medical University, Luzhou, Sichuan, China; ^2^ Department of Obstetrics, The First Affiliated Hospital of Chongqing Medical University, Chongqing, China; ^3^ Department of Radiation Oncology, Cancer Center, West China Hospital, Sichuan University, Chengdu, Sichuan, China

**Keywords:** cervical carcinoma, multiple metastases, interstitial brachytherapy, anlotinib, case report

## Abstract

**Background:**

Treatment of metastatic cervical cancer is a tricky issue. Currently, the National Comprehensive Cancer Network (NCCN) guideline recommends chemotherapy combined with bevacizumab for recurrent or metastatic cervical cancer. Still, the recurrence rate is high and the survival rate is low after standard treatment. We urgently need to achieve a multimodal therapy approach for recurrent or metastatic cervical cancer.

**Case description:**

We report the case of a patient with stage IB2 cervical squamous carcinoma who developed multiple metastases within a short term after receiving first-line standard treatment, and she underwent interstitial brachytherapy after systemic therapy with an encouraging outcome. The patient developed suspected inguinal lymph node metastases after 9 months at the end of first-line therapy and multiple metastases in the inguinal lymph nodes, anterior abdominal wall, and right lung after 17 months. As the patient had residual inguinal lymph nodes after systemic therapy, she received 3D-printed template-guided interstitial brachytherapy to the inguinal lymph nodes and maintenance therapy. By Sep 2023, she had achieved a good treatment outcome with a progression-free survival (PFS) of 36 months.

**Conclusion:**

Based on our patient response, when multiple metastases develop in the short term in early-stage cervical squamous carcinoma after first-line therapy, we may consider implementing local therapy combined with systemic therapy.

## Introduction

Cervical carcinoma (CC) is the fourth most common cancer and the leading cause of cancer deaths in women worldwide. Once cervical cancer recurs or metastasizes, the overall prognosis of patients is poor, with a 5-year survival rate of only 17%, a median survival time of 8-13 months, and overall survival (OS) of about 13-17 months (1, 2). Guidelines from the NCCN and the European Society of Gynecologic Oncology (ESGO) recommend palliative chemotherapy as the standard of care for patients with recurrent or metastatic disease (2, 3). However, the majority of patients who received palliative chemotherapy did not respond, and only about 1/4 of patients had a treatment response (4). Currently, the primary recommended regimen for first-line treatment of recurrent or metastatic cervical cancer is a triplet of chemotherapy combined with the anti-angiogenic drug bevacizumab, including cisplatin/paclitaxel/bevacizumab or carboplatin/paclitaxel/bevacizumab (3). The GOG-240 clinical trial showed that the addition of bevacizumab to chemotherapy prolonged the OS of patients, but only by 3.5 months (5).

Lymph node metastasis (LNM) is the most important independent risk factor affecting patient prognosis and leading to poor survival outcomes. LNM is an important route for cervical cancer spread. It is well known that the parametrial and obturator foramen lymph nodes are most likely to be involved in cervical cancer patients, followed by the internal, external, and common iliac lymph nodes, while the abdominal aorta and inguinal lymph nodes are relatively less frequently involved. Currently, reports on lymph node metastasis of cervical cancer are mainly focused on pelvic lymph nodes, and there are fewer reports on inguinal lymph node metastasis. It has been shown that even in patients who have completed pelvic lymph node dissection, 10% to 15% of patients initially considered N0 may develop recurrence or metastasis in the lymphatic area (6). Moreover, hematogenous metastasis is also a problem for advanced cervical cancer. Although hematogenous metastasis of cervical carcinoma is relatively uncommon, the common metastatic sites for advanced cervical cancer are the lung (36.3%), bone (16.3%), liver, and brain (7). It is rare for early-stage cervical cancer to develop lymph node metastasis or even hematogenous metastases shortly after the completion of radical surgery and postoperative adjuvant therapy.

Here we describe a case of a patient with cervical squamous carcinoma who developed inguinal lymph nodes, anterior abdominal wall, and right lung metastases shortly after undergoing first-line therapy. The patient underwent systemic treatment with chemotherapy and bevacizumab, leading to a complete response (CR) in the abdominal wall and lung, but only a partial response (PR) in the inguinal lymph nodes. Subsequently, the patient achieved an excellent outcome following interstitial brachytherapy and maintenance therapy with capecitabine and anlotinib.

## Case description

**Figure 1:**
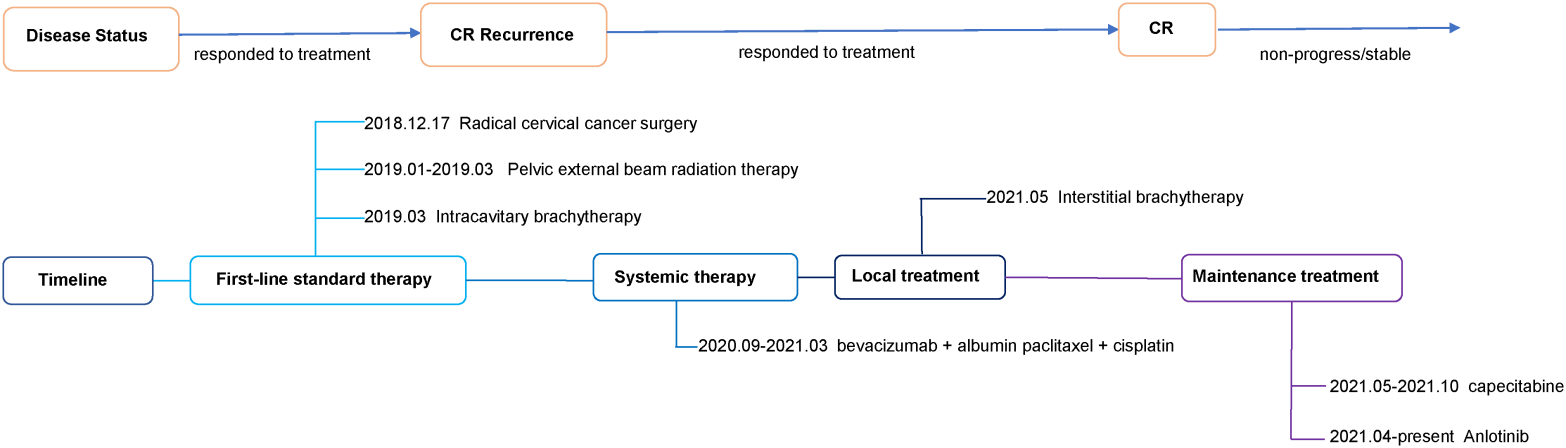
Timeline of different treatments and disease status.

A 42-year-old woman complained of abnormal vaginal discharge for over one month and was admitted to our hospital in Dec 2018 (Sichuan, China). We show the treatment timeline in **Figure 1**. She had 4 pregnancies, including 3 induced abortions and 1 spontaneous delivery. Prior personal and family history were negative. After the diagnosis of cervical cancer, the gynecologist performed a transabdominal extensive hysterectomy, bilateral salpingo-oophorectomy, and pelvic lymph node dissection under general anesthesia. Histopathological examination of the surgically obtained samples led to a squamous cell carcinoma of the cervix diagnosis. This pathological examination showed a tumor size of 4*2.5*2 cm with greater than 1/2 invasion of the cervical mesenchyme and vascular invasion. No metastasis were found in 12 left and 16 right pelvic lymph nodes. Pathological staging of surgery identified the disease as FIGO 2018 stage IB2.

According to Sedlis standards, the patient had intermediate risk factors for recurrence. The radiation oncologist performed postoperative adjuvant therapy. She underwent pelvic intensity-modulated radiotherapy (IMRT) (4510 cGy given in 25 fractions) for 5 weeks from Jan 2019 to Mar 2019. Subsequently, the patient received intracavitary brachytherapy with a prescription dose of 3000 cGy for 2 fractions. After treatment, follow-up magnetic resonance imaging (MRI) and squamous cell carcinoma antigen (SCCA) revealed no evidence of disease for 9 months.

In Dec 2019, the patient had an abnormal SCCA (36.78 ng/ml). Her MRI showed bilateral multiple inguinal lymph nodes, which were considered as possible metastases (enhanced partial circumferential enhancement). To further define the lymph nodes, the patient received a positron emission tomography-computed tomography (PET-CT). The result showed bilateral multiple inguinal lymph nodes, with some slightly enlarged, and no abnormalities in glucose metabolism. Combining the patient’s imaging and physical examination, the oncologist suggested continued follow-up. During the next 8 months of follow-up, the patient’s SCCA was abnormal but persistently decreasing (3.56-36.78 ng/ml), and MRI showed no significant progressive enlargement of the inguinal lymph nodes.

**Figure 2:**
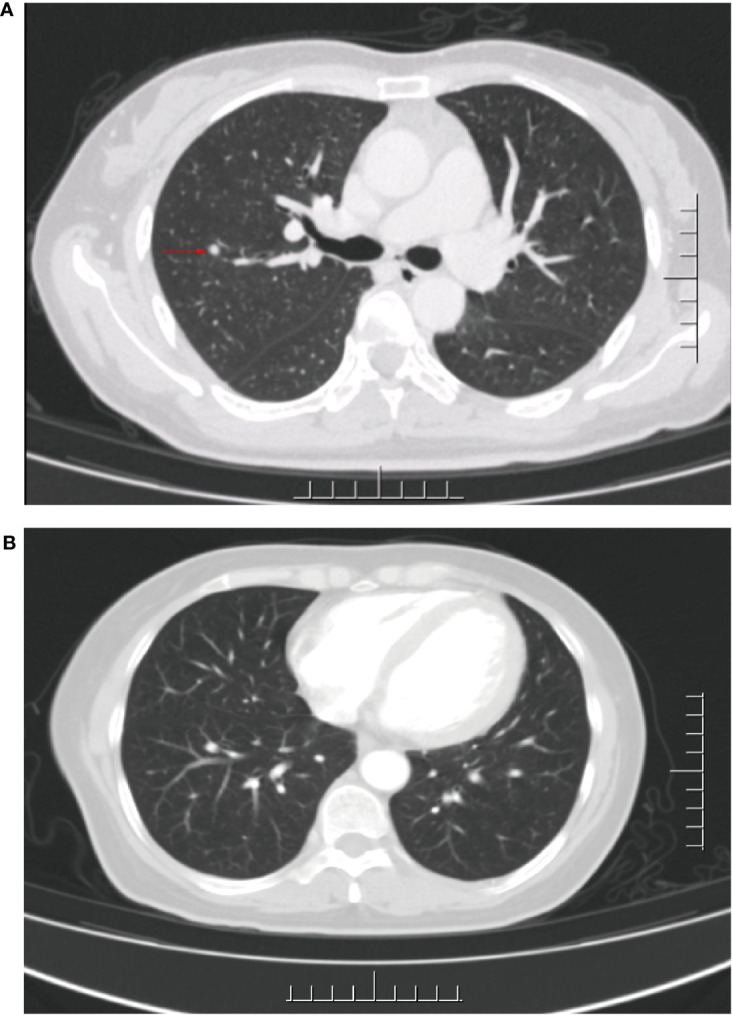
CT of the lungs before and after palliative chemotherapy. **(A)** CT after 17 months of first-line treatment: an approximately 4 mm diameter solid nodular shadow was seen in the right upper lung posterior segment. **(B)** CT after palliative chemotherapy: no significant shadows or nodules were seen in both lungs.

In Sep 2020, she visited our hospital for significantly enlarged bilateral inguinal masses. MRI showed additional anterior lower abdominal wall lesions, and bilateral enlarged and increased lymph nodes in the inguinal region (compared with the previous image). Cytologic aspiration biopsy confirmed the inguinal mass as metastatic carcinoma. Meanwhile, computed tomography (CT) showed a 4 mm solid nodule in the posterior segment of the upper lobe of the right lung (small nodule of 4 mm, not amenable to biopsy) (**Figure 2**). Based on the above clinical results, we considered that our patient had inguinal lymph nodes, anterior abdominal wall, and right lung metastases after postoperative radiotherapy for stage IB2 cervical squamous cell carcinoma.

**Figure 3:**
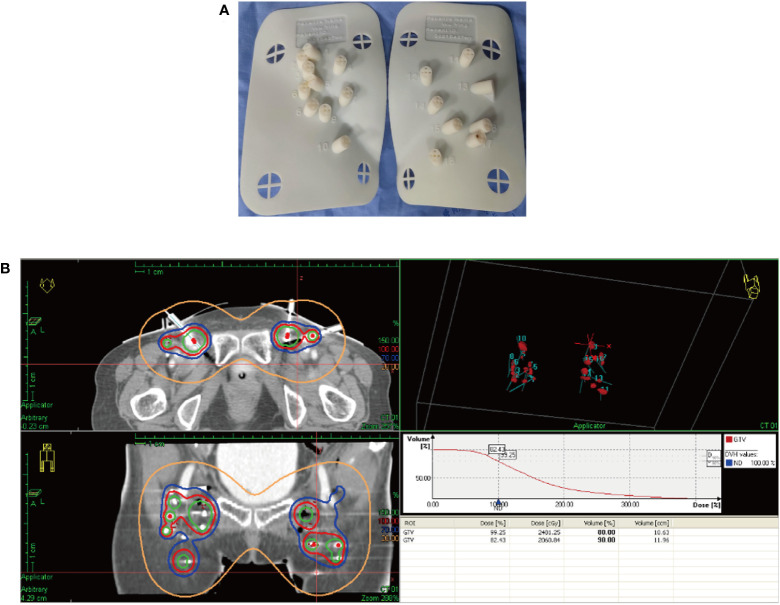
3D printed templates guide interstitial brachytherapy. **(A)** 3D printed molds of the patient’s inguinal region. **(B)** Three-dimensional conformal dose assessment.

After receiving 8 cycles of palliative chemotherapy and targeted therapy (bevacizumab, 7.5 mg/kg, d0 + albumin paclitaxel, 175 mg/m2, d1 + cisplatin, 75 mg/m2, d2-3, q21d) from Sep 2020 to Mar 2021, the patient achieved a CR in the abdominal wall metastasis and lung metastasis (**Figure 2**). Additionally, she achieved a partial response (PR+; regression of all lesions without complete resolution) in the inguinal lymph nodes. In May 2021, the radiation oncologist created 3D-printed templates to guide interstitial brachytherapy on the inguinal lymph node metastases, and the prescription dose was 2500cGy. This is a single hypofractionated radiotherapy that utilizes a high dose rate (HDR) of Ir-192 radioisotope for interstitial brachytherapy. we performed it using a microSelectron v3 after-loader (Elekta, Holland) with a planning system of oncentra 4.5. **Figure 3** shows the 3D printed molds and three-dimensional conformal dose assessment for interstitial brachytherapy. MRI and CT 1 month later confirmed a PR.

**Figure 4:**
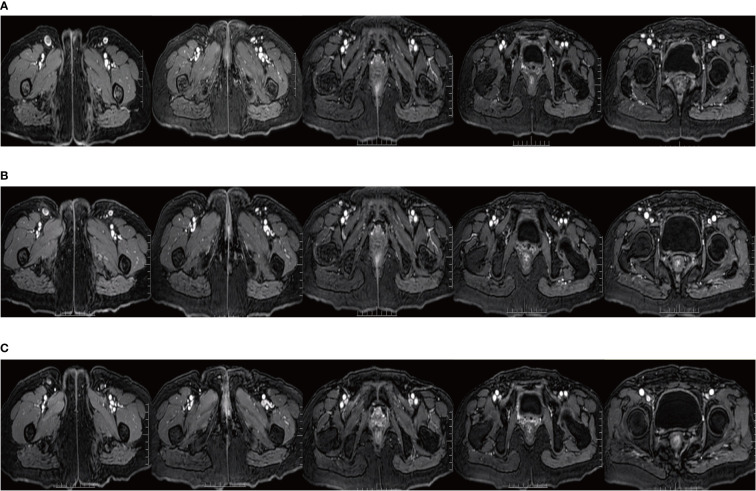
MRI of the inguinal region before and after interstitial brachytherapy. **(A)** MRI at the end of the palliative chemotherapy. **(B)** MRI of the inguinal region 3 months after interstitial brachytherapy. **(C)** MRI of the inguinal region 1 year after interstitial brachytherapy.

Since Apr 2021, she has been started on the tyrosine kinase inhibitor (RTK) anlotinib (10 mg, qd) on the advice of the oncologist. From May 14, 2021 to Oct 8, 2021, she took a total of 7 cycles of the fluorouracil-based chemotherapy drug capecitabine (1250 mg/m2, bid, for 14 days with 7 days rest). In Sep 2021, her MRI showed shrinking inguinal lymph nodes. We considered that the patient’s inguinal lymph nodes remained stable during maintenance therapy. During maintenance therapy with capecitabine and anlotinib, her serum SCCA levels have remained low and stable (0.91-2.79 ng/ml). Considering the relatively great toxicity of dual-agent combination maintenance therapy and the patient’s personal preference, she subsequently chose to remain on single-agent maintenance therapy with anlotinib. Her latest serum SCCA was 1.01 ng/ml (Sep 20, 2023). Meanwhile, follow-up MRI (from Jun 16, 2021, to Sep 20, 2023) confirmed that the bilateral inguinal lymph nodes had not shown progression after showing shrinkage. **Figure 4** shows the MRI of the patient before and after interstitial brachytherapy in the inguinal region. After radiotherapy, the patient had slight localized redness and swelling. The patient now has no skin ulcers, no bilateral lower extremity edema or other complications, and only mild localized skin pigmentation. As of Sep 2023, the PFS reached 36 months.

During maintenance therapy, the patient experienced adverse events (AEs) of hypertension and hand-foot syndrome (HFS). The patient’s blood pressure has ranged from 109-143/78-102 mmHg. Currently, the patient is taking the oral calcium channel blocker amlodipine to treat hypertension, and her blood pressure is now well controlled. Since Sep 2021, the patient has been experiencing grade 3 HFS with desquamation, but no blistering or bleeding. The patient has been using topical medications to alleviate this adverse reaction, and her skin is currently in fair condition.

## Discussion

There are few effective therapeutic options for recurrent or metastatic cervical cancer. Currently, the primary recommended regimen for first-line treatment of recurrent or metastatic cervical cancer is a triplet of chemotherapy combined with the anti-angiogenic drug bevacizumab, either cisplatin/paclitaxel/bevacizumab or carboplatin/paclitaxel/bevacizumab (3). This is since the addition of bevacizumab to chemotherapy was shown to prolong OS and PFS in a clinical trial (GOG240) (5). Our patient developed multiple metastases to the inguinal lymph nodes, anterior abdominal wall, and right lung shortly. Considering the patient’s condition and clinical practice, we also followed the guidelines and chose chemotherapy combined with bevacizumab as the primary treatment option. However, our patient still had residual lesions. Studies have shown that while bevacizumab has shown a survival benefit in first-line treatment of recurrent or metastatic cervical cancer, the OS benefit is limited and the response is short-lived (5, 8). This is consistent with the patient achieving only a partial response after 8 cycles of systemic therapy. Based on the Phase III Keynote 826 study, the latest NCCN guidelines include the programmed death ligand 1 (PD-L1) inhibitor pembrolizumab + chemotherapy ± bevacizumab in the first-line treatment recommendation (3, 9). In future clinical practice, we can consider immunotherapy as a priority after determining patients’ PD-L1 status. Our patient declined our proposed PD-L1 test for financial reasons.

The NCCN guideline recommends surgical resection and/or local external beam radiation therapy and/or local ablation for patients with recurrent or metastatic cervical cancer who can be treated locally (3, 10, 11). Reports on response rates to local therapy for metastatic cervical cancer are relatively rare. Studies have shown that once recurrence or metastasis occurs, it is difficult to obtain clinical benefit through radical resection and local radiotherapy (12, 13). However, studies have confirmed that oligometastatic cervical cancer patients with lymph node, lung, liver, or bone metastases may benefit from aggressive local therapy (14, 15). A retrospective study included 50 cases of distant metastatic cervical cancer, including 6 patients with only inguinal lymph node metastases, and confirms that individualized local therapy can achieve good results (14). Our patient still had residual inguinal lymph nodes after 8 cycles of systemic therapy. The clinical oncologists at our hospital considered a local treatment option for the patient. Firstly, we were concerned about potential postoperative complications, such as lower extremity edema and urinary retention, which could significantly impact the patient’s quality of life. Secondly, the patient had a relatively large number of metastatic lymph nodes in the bilateral inguinal region, making local ablative therapy unfeasible. Lastly, the patient had previously undergone pelvic external beam radiation therapy, which posed challenges for re-external irradiation due to the presence of a low-dose area of radiation in the inguinal region. So, we decided to perform bilateral inguinal lymph node interstitial brachytherapy for the patient.

In clinical practice, advanced metastatic cervical cancer enters maintenance therapy or follow-up after the completion of systemic therapy. However, the response rate for second-line or follow-up treatment is relatively low (15%-30%) and the duration of response for recurrent or metastatic cervical cancer is short (2). This is also the reason why we choose to perform local therapy after systemic treatment. We hope that by improving the local control rate and thus possibly translating into a survival benefit, the patient’s PFS has reached 36 months today. More surprisingly, our patient experienced only localized redness after completing interstitial brachytherapy. At the follow-up, she only complained of slight local skin pigmentation and no adverse effects such as bilateral lower limb edema or skin breakdown. This suggests that the local treatment modality of interstitial brachytherapy not only may translate into a survival benefit, but also greatly improves the quality of life of the patient.

We treated the patient with maintenance therapy hoping to achieve a better outcome. Studies have confirmed that 5-fluorouracil drugs are effective in recurrent or metastatic cervical cancer (16, 17). However, the therapeutic efficacy of anlotinib in cervical cancer is not clear. In China, a single-arm prospective II clinical trial evaluating the efficacy and safety of anlotinib in patients with recurrent or metastatic cervical cancer demonstrated that at 16-month follow-up, patients treated with anlotinib had a median progression-free survival (mPFS) of 3.2 months, a median overall survival (mOS) of 9.9 months, an overall objective remission rate (ORR%) of 24.4%, and a disease control rate (DCR%) of 58.5% (18). Although the use of anlotinib as maintenance therapy is not yet in the guidelines, the above research findings provide a clinical basis for our choice of anlotinib as a maintenance phase of therapy. During maintenance therapy, the patient had stable disease. This outcome is very encouraging.

We were pleasantly surprised to find that interstitial radiation therapy for cervical cancer in the presence of inguinal lymph node metastases can lead to better treatment outcomes. It is worth noting that the maintenance therapy with anlotinib might also be one of the reasons for the promising outcome of the patient. Further investigation is required to explore the underlying mechanism.

## Conclusion

In conclusion, it is very rare for early-stage cervical squamous carcinoma to develop multiple metastases shortly after first-line treatment, particularly inguinal lymph node metastases following pelvic external beam radiation therapy. In our case, the patient received inguinal lymph node interstitial brachytherapy and maintenance therapy after systemic treatment showed a remarkable clinical response. Moreover, the patient’s AEs were only mild HFS and mild hypertension. The encouraging outcome should be confirmed in clinical practice dealing with similar patients.

## Data availability statement

The original contributions presented in the study are included in the article/supplementary material. Further inquiries can be directed to the corresponding authors.

## Ethics statement

Written informed consent was obtained from the individual(s) for the publication of any potentially identifiable images or data included in this article.

## Author contributions

YQ: Visualization, Writing – original draft. PG: Writing – original draft. DL: Writing – review & editing. HH: Writing – review & editing. WH: Writing – review & editing. LT: Writing – review & editing. XD: Writing – review & editing. BL: Supervision, Writing – review & editing. QW: Supervision, Writing – review & editing. ZZ: Conceptualization, Supervision, Writing – review & editing.
